# Longitudinal clinical observation of 4 patients with preclinical stage of idiopathic normal pressure hydrocephalus

**DOI:** 10.1186/2045-8118-12-S1-P4

**Published:** 2015-09-18

**Authors:** Shingo Azuma, Takashi Suehiro, Hiroaki Kazui, Shunsuke Sato, Yukiko Suzuki, Hideki Kanemoto, Kenji Yoshiyama

**Affiliations:** 1 Osaka University Graduate School of Medicine, Japan

## Introduction

In patients with idiopathic normal pressure hydrocephalus (iNPH) with features of Disproportionately Enlarged Subarachnoid-space Hydrocephalus (DESH), ventriculomegaly and the tight high-convexity and medial subarachnoid spaces appear in magnetic resonance (MR) images before the occurrence of objective symptoms. There are few reports of longitudinal observation of clinical course in DESH-type iNPH patients with no objective symptom.

## Methods

We longitudinally observed 4 patients (1 female and 3 males with a mean age of 73.8 years) with features of DESH who visited the neuropsychological clinic in our hospital, whose symptoms were not apparent in the first visit. We evaluated the triad symptoms with iNPHGS and conducted the cognitive and gait examinations once a year. We also evaluated quantitative rCBF of those patients by 123I-IMP single photon emission computed tomography (SPECT) using the autoradiography (ARG) method.

## Results

Based on the scores of iNPHGS, we classified 4 patients into two groups; two stable patients and two deteriorated patients. In one patient of the stable group, the score of the Mini-mental State Examination (MMSE) did not change and the score of the Frontal Assessment Battery (FAB) and the Timed Up & Go Test (TUG) improved, however, in the other patient, the score of MMSE, FAB and TUG worsened. In the deteriorated group, one patient expressed the gait disturbance and the other patient did the gate disturbance and the cognitive impairment. In the former patient, the score of MMSE and FAB worsened and a wide-based gate and the disturbance of the dynamic equilibrium appeared, although the score of TUG did not change. In the latter patient, the score of FAB and TUG worsened but the score of MMSE did not change. The changes of quantitative rCBF of 4 patients were various.

## Conclusions

The different clinical courses and changes of quantitative rCBF were observed in each patient.

